# Protective Role of Somatostatin in Sepsis-Induced Intestinal Barrier Dysfunction through Inhibiting the Activation of NF-*κ*B Pathway

**DOI:** 10.1155/2020/2549486

**Published:** 2020-12-10

**Authors:** Xin Xu, Quanli Zhu, Guoliang Li, Junjian Ma, Zhijian Pan, Wei Wu

**Affiliations:** Department of Gastrointestinal Hepatobiliary Surgery, The Affiliated Hospital of Hangzhou Normal University, Gongshu, Hangzhou 310015, China

## Abstract

Somatostatin (SST) has a protective role in intestinal injury, inflammatory response, and intestinal mucosal barrier in rats with acute pancreatitis. However, its function in sepsis-induced intestinal barrier dysfunction remains largely unknown. A mouse sepsis model was constructed, and SST was injected into the tail vein. Then, hematoxylin and eosin staining (HE) was used to detect the intestinal barrier dysfunction. Enzyme-linked immunosorbent assay was used to detect the level of tumor necrosis factor *α*- (TNF-) *α*, interleukin- (IL-) 6, and interleukin- (IL-) 10 in the ileum. Expressions of tight junction proteins, zonula occludens- (ZO-) 1 and Claudin-1, and NF-*κ*B p65 in the ileum were detected using western blot and immunohistochemistry as needed. Furthermore, JSH-23 as an inhibitor of the NF-*κ*B pathway was injected into sepsis mice with SST or not. Mice with sepsis showed an obvious intestinal barrier dysfunction with decreasing specific somatostatin receptor subtype (SSTRs), and increasing TNF-*α*, IL-6, and IL-10 in the ileum. SST could relieve the injury, the decrease of SSTRs, and the increase of TNF-*α* and IL-6 induced by sepsis and also further enhanced the expression of IL-10. Further analysis showed that ZO-1 and Claudin-1 were reduced in the ileum by sepsis but enhanced by SST. NF-*κ*B p65 was promoted in the ileum by sepsis but inhibited by SST. Further experiments confirmed that NF-*κ*B inhibitor JSH-23 could repair the intestinal barrier dysfunction and enhance the protective effect of SST on the intestinal barrier. SST, with a protective effect on intestinal barrier dysfunction through suppression of NF-*κ*B, could be a potential therapeutic drug for sepsis-induced intestinal barrier dysfunction.

## 1. Introduction

Sepsis is a systemic abnormality caused by a dysregulated host response to infection and characterized by immune activation and organ dysfunction [1]. Sepsis is one of the common severe complications of severe trauma, burn, infection, and other clinically critical patients [2–4]. It is noted that patients with sepsis have risk of progressing to severe sepsis or septic shock in a short time, which is a deadly condition with significant morbidity [5].

Normal intestinal mucosal barrier as the physical and metabolic barrier can effectively prevent intestinal bacteria and other harmful substances from invading the body's nonintestinal aseptic tissue [6]. Sepsis can cause edema and damage of intestinal mucosa epithelial cells [7] and the increase of intestinal permeability and intestinal barrier dysfunction, which in turn lead to bacteria and endotoxin in the gut and the migration of harmful contents, causing severe systemic inflammatory response, increasing sepsis condition, and further damaging the intestinal barrier function [8, 9]. Therefore, the protection of intestinal barrier function is the key to treat sepsis and improve the progress of sepsis.

Somatostatin (SST) is a neuroendocrine polypeptide, which is mainly used to treat acute pancreatitis [10], portal hypertension [11], gastrointestinal bleeding [12], and endocrine diseases [13]. At present, increasing basic and clinical studies have shown that SST can inhibit inflammatory response [14], oxidative damage [15], cell protection, and immune regulation [16]. Besides, the role of SST in intestinal inflammatory has been reported. For instance, the proliferation of immature B cells is involved in intestinal injury induced by intestinal ischemia-reperfusion, while SST greatly improves B cell maturity in macaques and thereby limits intestinal injury [17]. Another report shows that SST protects intestinal ischemia-reperfusion-induced gut injury through suppression of gut-derived toxic mediators reaching systemic circulation and increases of the number of lymphocyte homing to the effector sites [18]. The combination of the pharmacological action of SST and the existing animal experiment results indicates that SST may have a certain protective effect in dealing with the functional impairment of the intestinal mucosal barrier [19]. However, the effect of SST on intestinal barrier dysfunction induced by sepsis has not been recognized.

Nuclear factor kappa B (NF-*κ*B) is a transcriptional factor involved in the regulation of the expression of multiple immune or inflammatory genes [20, 21]. NF-*κ*B usually remains inactive in the cytoplasm through association with the inhibitor I*κ*B. The activation of the inhibitor of *κ*B kinase (IKK) complex induces phosphorylation of I*κ*B molecules and degradation and thereby release of NF-*κ*B, which translocates to the nucleus to promote gene transcription [22]. NF-*κ*B plays a central role in regulating the transcription of several genes associated with sepsis/septic shock [23]. It is known that the activation of the NF-*κ*B/HIF-1*α* pathway involves intestinal barrier dysfunction [24]. A previous research shows that the SST derivate (smsDX) suppresses NF-*κ*B expression to attenuate tumor-associated macrophage-stimulated progress of prostate cancer [25]. A previous research finds that the decrease of NF-*κ*B nuclear expression has been detected in SST-treated macaques with intestinal ischemia-reperfusion injury [26]. However, the function of NF-*κ*B in intestinal barrier dysfunction has not been illuminated well.

Therefore, a sepsis mouse model was constructed, and the effect of SST on intestinal barrier dysfunction in CLP-induced septic mouse was assessed. We aimed to investigate the role and the underlying mechanism of SST in sepsis-induced intestinal barrier dysfunction.

## 2. Methods

### 2.1. Ethic Statement

Animal experiments in this research were conducted following the guidelines of China Council on Animal Care and Use. This study has obtained approval from the Committee of Experimental Animals of the Affiliated Hospital of Hangzhou Normal University (No. C20170813007M). Efforts were made to minimize the pain and discomfort to the animals. All animal experiments were conducted in the Affiliated Hospital of Hangzhou Normal University.

### 2.2. Animal Model Construction

One hundred male C57BL/6 mice (10 weeks, 20-25 g) were purchased from HFK Bioscience, Beijing, China. Mice were kept in a standardized temperature (25°C-28°C), humidity (50-60%), and light environment (12 h light/12 h dark) and fed with standard food and tap water for one week. For sepsis model construction, cecal ligation and puncture (CLP) operation was performed [27]. Briefly, mice inhaled isoflurane for anesthesia during the whole course. After abdominal sterilization, the abdominal cavity was opened in the middle of the abdomen, and the cecum was found with tweezers. The cecum was gently pulled out of the abdominal cavity and ligated with aseptic 3-0 suture 1 cm from the end of the cecum. The 22 G needle was inserted twice in an area with fewer blood vessels. Then, the cecum was gently squeezed to make sure the puncture site is clear. Then, the extracted segment was inserted into the abdominal cavity and the peritoneum, and abdominal muscles were closed with aseptic suture 6-0; lidocaine was placed on the incision for pain relief, and then, the skin was sutured. Mice in the sham group underwent the same operations except for cecal ligation and perforation. After the operation, 1 mL of normal saline was added to the back of the mice, which were observed in the cage for one hour and then returned to the animal feeding center.

### 2.3. Animal Grouping

To investigate the function of SST in sepsis-induced intestinal barrier dysfunction, SST (P9077, Beyotime, Shanghai, China) was purchased and diluted into DMSO. Forty mice were randomly divided into four groups: control group (no operation, *n* = 10); sham groups (no cecal ligation and perforation, *n* = 10); model group (CLP operation, *n* = 10); and model+SST (CLP operation combined with SST injection (5 *μ*g/kg/h), *n* = 10).

To explore the role of NF-*κ*B in sepsis-induced intestinal barrier dysfunction, sixty mice were randomly divided into six groups: control group (no operation, *n* = 10); sham groups (no cecal ligation and perforation, *n* = 10); model group (CLP operation, *n* = 10); model+SST (CLP operation combined with SST injection (5 *μ*g/kg/h), *n* = 10); model+SST+JSH-23 (CLP operation combined with SST and JSH-23 injection, 50 mg/kg, *n* = 10); and model+JSH-23 (CLP operation combined with JSH-23 injection, 50 mg/kg, *n* = 10). At 48 h after CLP operation, all mice were euthanized by an overdose intravenous injection of isoflurane (5%, Sigma-Aldrich, USA). The pathological damage analysis was performed immediately. JSH-23 was an NF-*κ*B inhibitor bought from Calbiochem-EMD Biosciences, Inc. (San Diego, CA, USA).

### 2.4. Hematoxylin and Eosin (HE) Staining

The ileum tissues were fixed in 4% formaldehyde and embedded in paraffin wax using standard techniques. Slices (5 *μ*m) were cut and stained with hemotoxylin for 10 min and eosin (C0105, Beyotime, China) for 30 s at room temperature. Then, the slices were washed by 70% ethyl alcohol twice.

### 2.5. Measurement of Transepithelial Electrical Resistance (TER)

Intestinal mucosae dissected from the muscular layer were immediately kept in all sides of an Ussing chamber (Physiologic Instrument, USA) after animals were sacrificed. The spontaneous potential difference (PD) was maintained at a level of 0 mV. The short-circuit current (*Δ*Isc) was measured by applying 1 mV pulse for 1 s at 60 s intervals. TER was calculated by TER (*Ω*∙cm^2^) = PD/ΔIsc.

### 2.6. Enzyme-Linked Immunosorbent Assay (ELISA)

The ileum samples were homogenated at 4°C, and then, the homogenate was added into a 96-well ELISA plate. To measure the concentrations of the inflammation-related cytokines, TNF-*α* (CAT: PT512), IL-6 (CAT: PI326), and IL-10 (CAT: PI522), ELISA kits (Beyotime, China) were used and experiments were performed according to the introduction. The absorption value at 450 nm was read on a SpectraMax Plus 384 Microplate Reader (Plus 384, Molecular Devices, USA). Each sample was detected with 6 parallel repeats.

### 2.7. Western Blot

The total proteins in ileum tissues were extracted by Radio-Immunoprecipitation Assay (RIPA, 89901, Thermo Scientific, USA). The protein concentration was detected by the BCA method, and the proteins (25 *μ*g in each lane) were electrophoresed in 6% polyacrylamide gel. The isolated proteins were then transferred onto a PVDF membrane. The membrane was blocked for 1 h at room temperature for nonspecific binding with 5% bovine serum albumin (BSA). After that, the membrane was incubated with rabbit anti-zonula occludens- (ZO-) 1 antibody (1 : 1000, ab96587, Abcam, UK), rabbit anti-Claudin-1 antibody (1 : 1000, ab15098, Abcam, UK), anti-NF-*κ*B p65 (1 : 5000, ab207297, Abcam, UK), and rabbit anti-GAPDH antibody (1 : 1000, ab181602, Abcam, UK) at 4°C for the night. The membrane was exposed into Goat Anti-Rabbit IgG H&L (1 : 5000, ab207297, Abcam, UK) at room temperature for 1 h, and blots were performed using ECL detection reagents (Merck Millipore, USA).

### 2.8. Immunohistochemistry Staining

Briefly, paraffin sections were dewaxed and incubated with 3% hydrogen peroxide (H2O2) to block the endogenous peroxidase activity. Pepsin K was applied following the exposure to anti-NF-*κ*B p65 antibody (1 : 1000, ab207297, Abcam, UK) at 4°C overnight. After incubation with the HRP-conjugated secondary antibody (Goat Anti-Rabbit IgG H&L, ab205718, Abcam, UK), DAB (ab64238, Abcam, UK) was used for immunohistochemical staining. The slides were then dehydrated in graded alcohol and sealed. The images were acquired under the light microscopy (TS100, Nikon, Japan).

### 2.9. Quantitative Real-Time Polymerase Chain Reaction (qRT-PCR)

The total RNA in tissues was extracted using the TRIzol method. Briefly, the RNA was separated from tissues using 1 mL TRIzol (Invitrogen, USA). The extracted total RNA was reverse-transcribed into cDNA by applying TaKaRa PrimeScript ™ RT Reagent Kit (TaKaRa, Japan). QRT-PCR was conducted in the Thermo Fisher Scientific real-time PCR system (Thermo Fisher, USA) under the following conditions: predenaturation at 95°C for 10 minutes (min), denaturation at 95°C for 15 seconds (s), and annealing at 60°C for 1 min, for a total of 35 cycles. The sequences of the primers synthesized by Sangon Biotech (Shanghai, China) are listed below: SSTR5-F, 5′-TGGTCTTTGCGGATGTCCAGGA-3′, and SSTR5-R, 5′-CAAAGAAGCCCAGCACAGACGT-3′; GAPDH (internal reference)-F, 5′-GAT GCT GGT GCT GAG TAT GRC G-3′, and GAPDH-R, 5′-GTG GTG CAG GAT GCA TTG CT CTG A-3′.

### 2.10. Statistical Analysis

All quantitative data were presented as mean ± SD from the five animals in each group. Duplicate measurements were made for each animal and were analyzed using the SPSS (version 10.0; SPSS, Inc., Cary, NC, USA). The data were evaluated with ANOVA then confirmed by a post hoc test for multiple comparisons. Significance was set at *P* < 0.05.

## 3. Results

### 3.1. SST Prevented Intestinal Barrier Dysfunction in Mice with Sepsis

**Figure 1:**
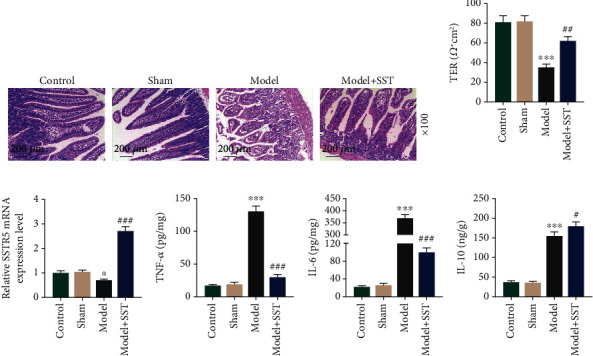
SST prevented sepsis-induced intestinal barrier dysfunction. (a) Intestinal tissue from the control, sham, model, and model+SST group was detected using hematoxylin and eosin (HE) staining. Sections presented ten mice in each group. Magnification: 100x. Scale bar: 200 *μ*m. (b) TER of gut mucosae was evaluated via transepithelial voltohmmeter. (c) The specific somatostatin receptor subtype SSTR5 was detected by qRT-PCR. (d–f) Detection of inflammatory factors, including TNF-*α*, IL-6, and IL-10 in the ileum using ELISA. Data were shown as the mean ± SD from 3 independent experiments. ^∗∗∗^*P* < 0.001, vs. sham; ^###^*P* < 0.001, vs. model. TNF-*α*: tumor necrosis factor *α*; IL-6: interleukin 6; IL-10: interleukin 10; SST: somatostatin.

To evaluate the effect of SST on intestinal barrier function in mice with sepsis, HE staining was performed and we found that the intestinal mucosa in the model group appeared atrophic, with shorter intestinal villi and disordered arrangement and obvious gap between intestinal villous epithelium, while SST could obviously prevent the intestinal barrier dysfunction (Figure 1(a)). To clarify whether SST affects gut barrier permeability, TER of the gut epithelium were examined, in which the mice with CLP had a significant decrease of TER, while SST increased the TER of the model group (Figure 1(b)). The mRNA expression of specific somatostatin receptor subtype SSTR5 was discovered decreased in the model group but enhanced by SST (Figure 1(c)). Furthermore, the contents of TNF-*α*, IL-6, and IL-10 were detected in order to study the inflammation in mice with sepsis. ELISA analysis indicated that inflammatory factors, TNF-*α* and IL-6, and anti-inflammatory factor, IL-10, were all promoted in the ileum by sepsis, while SST treatment could significantly weaken the increase of TNF-*α* and IL-6 and further enhanced the IL-10 levels (Figures 1(d)–1(f), *P* < 0.001).

### 3.2. SST Prevented the Reduction of Tight Junction Protein Expressions and the Activation of NF-*κ*B p65

**Figure 2:**
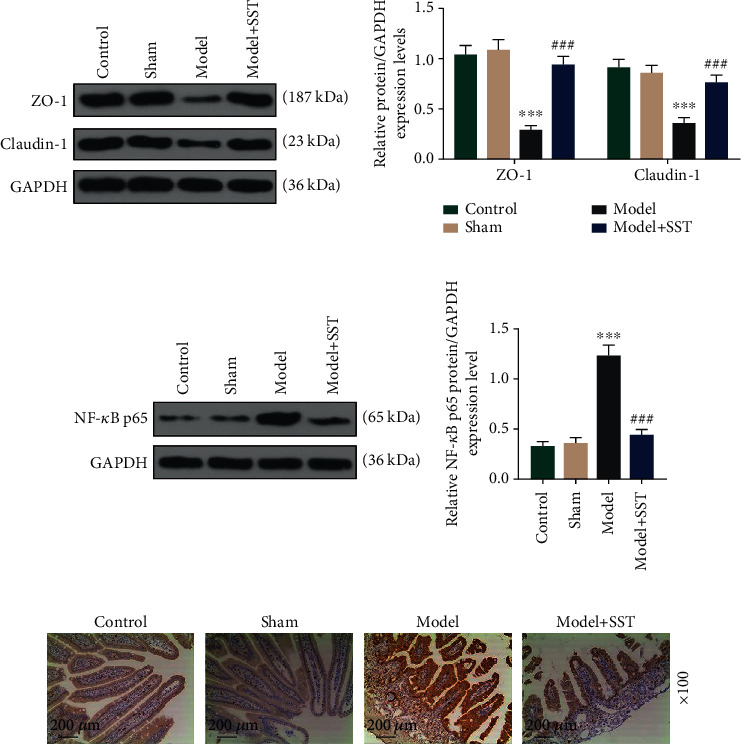
SST prevented the reduction of tight junction protein expressions and the activation of NF-*κ*B p65: (a) western blot analysis of tight junction proteins, ZO-1 and Claudin-1, in the ileum; (b, c) western blot and immunohistochemistry analysis of NF-*κ*B p65 in the ileum. Magnification: 100x. Scale bar: 200 *μ*m. Data were shown as the mean ± SD from 3 independent experiments. ^∗∗∗^*P* < 0.001, vs. sham; ^###^*P* < 0.001, vs. model. ZO-1: zonula occludens 1; SST: somatostatin.

ZO-1 and Claudin-1 are tight junction proteins. To explore the effect of SST on intestinal barrier dysfunction, expressions of ZO-1 and Claudin-1 were detected using western blot. The result indicated that ZO-1 and Claudin-1 were reduced in mice with sepsis, while decrease of ZO-1 and Claudin-1 expressions could be reversed by SST treatment (Figure 2(a), *P* < 0.001). NF-*κ*B is closely associated with the progress of sepsis and sepsis-induced intestinal barrier dysfunction [28]; thus, we were interested in that whether the NF-*κ*B pathway could be regulated by SST. Results from western blot and immunohistochemistry indicated that NF-*κ*B p65 was promoted by sepsis while inhibited by SST (Figures 2(b) and 2(c), *P* < 0.001).

### 3.3. JSH-23 Enhanced the Protective Effect of SST on Sepsis-Induced Intestinal Barrier Dysfunction

**Figure 3:**
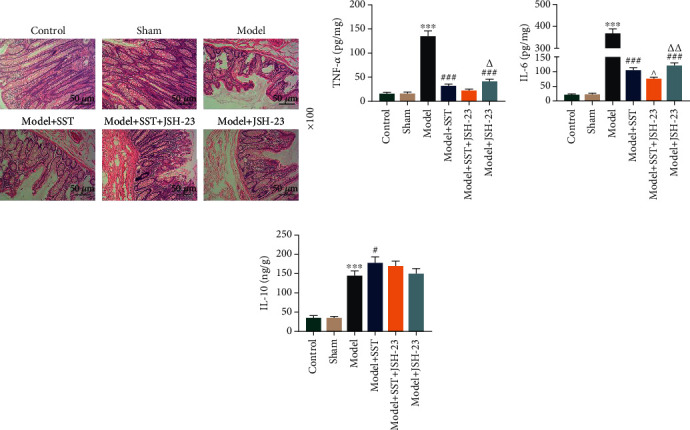
JSH-23 enhanced the protective effect of SST on sepsis-induced intestinal barrier dysfunction. (a) Intestinal tissue from the control, sham, model, model+SST group, model+SST+JSH-23 group, and model+JSH-23 group was detected using hematoxylin and eosin (HE) staining. Sections presented ten mice in each group. Magnification: 100x. Scale bar: 200 *μ*m. (b–d) Detection of inflammatory factors, including TNF-*α*, IL-6, and IL-10, in the ileum using ELISA. Data were shown as the mean ± SD from 3 independent experiments. ^∗∗∗^*P* < 0.001, vs. sham; ^#^*P* < 0.05; ^###^*P* < 0.001, vs. model. ^^^*P* <0.05, vs. model+SST. ^△^*P* <0.05, ^△△^*P* < 0.01, vs. model+SST+JSH-23. SST: somatostatin.

JSH-23 is a well-known NF-*κ*B p65 inhibitor. To investigate the function of NF-*κ*B p65 in SST-treated model mice, JSH-23 was injected into mice and intestinal barrier dysfunction was assessed again. It was shown that intestinal mucosa injury induced by sepsis (model group) could be prevented by SST treatment, while JSH-23 protected mice from intestinal mucosa dysfunction and enhanced the protective effect of SST on the intestinal mucosa (Figure 3(a)). Further analysis indicated that JSH-23 decreased the levels of TNF-*α*, IL-6, and IL-10 in the ileum from mice with sepsis and enhanced the suppressive effect of SST on TNF-*α* and IL-6 and meanwhile enhanced the IL-10 level (Figures 3(b)–3(d), *P* < 0.05).

### 3.4. JSH-23 Repaired the Decrease of Sepsis-Induced Tight Junction Protein Expressions and Blocked the Effect of SST

**Figure 4:**
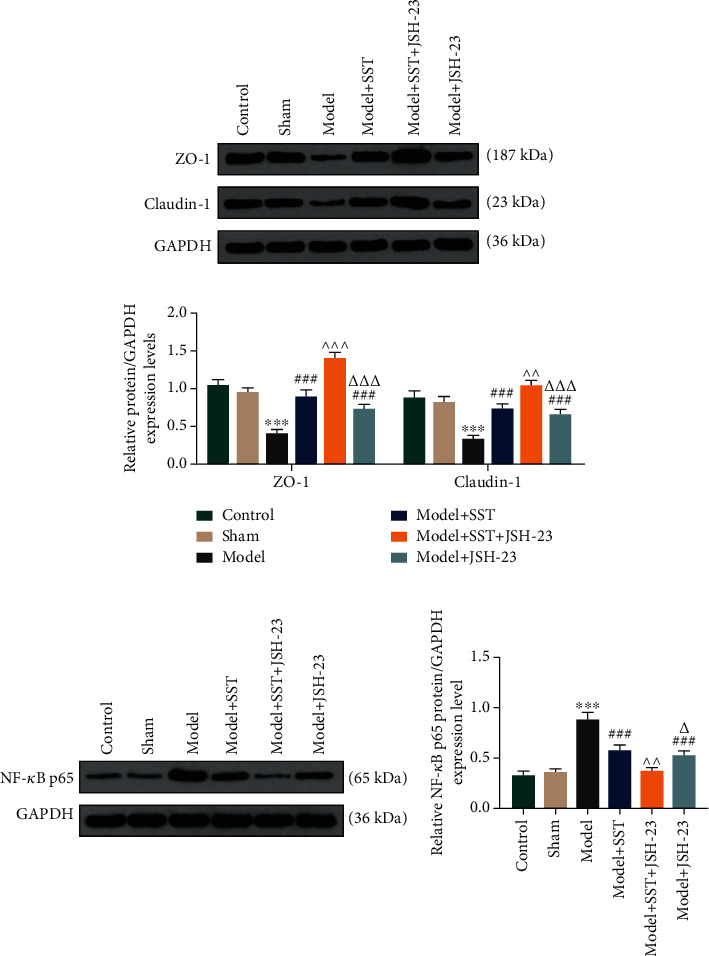
JSH-23 enhanced the protective effect of SST on the decrease of sepsis-induced tight junction protein expressions: (a) western blot analysis of tight junction proteins, ZO-1 and Claudin-1, in the ileum; (b) western blot analysis of NF-*κ*B p65 in the ileum. Data were shown as the mean ± SD from 3 independent experiments. ^∗∗∗^*P* < 0.001, vs. sham; ^###^*P* < 0.001, vs. model. ^^^^*P* < 0.01 and ^^^^^*P* < 0.001, vs. model+SST. ^△^*P* < 0.05 and ^△△△^*P* < 0.001, vs. model+SST+JSH-23. SST: somatostatin.

Next, we analyzed the levels of tight junction protein expressions in different groups. Western blot analysis showed that sepsis-induced reduction of ZO-1 and Claudin-1 in the ileum was blocked by SST or/and JSH-23 (Figure 4(a), *P* < 0.05). We also found that NF-*κ*B p65 promoted by sepsis was blocked by SST or/and JSH-23 (Figure 4(b), *P* < 0.05). Those results suggested that the protective role of SST in sepsis-induced intestinal barrier dysfunction might be mediated by the NF-*κ*B pathway.

## 4. Discussion

In our research, we found that SST had a protective role in sepsis-induced intestinal barrier dysfunction. And inhibiting the activation of the NF-*κ*B pathway may be involved in this progress.

CLP-induced sepsis model refers to the body's defense response and systemic inflammation through the formation of trauma and local infection in animals. During model construction, overflow feces can enter the abdominal cavity through perforation, causing mixed infection of intestinal flora. Meanwhile, the ligated cecum is ischemic and necrotic, releasing inflammatory factors and inducing sepsis. IL-10 is a crucial anti-inflammatory cytokine and immune support factor and also mediates the downregulation of proinflammatory cytokines such as TNF-*α* and IL-6 at the early stage of sepsis [29]. In this study, we observed the severe inflammatory damage with increase of TNF-*α*, IL-6, and IL-10 in the ileum from the CLP-induced sepsis model, which was consistent with the previous study [30]. We consider that the increase of IL-10 in the sepsis mouse was realized to protect the host from tissue damage during acute phases of immune responses [31], and SST could further enhance this protective progress.

ZO-1 and Claudin-1 are both tight junction proteins. Claudin-1 is widely expressed in the intestinal epithelium and is also known by its barrier-forming abilities and has been proposed to have an important role for tight junction integrity [32]. ZO-1 belongs to cytoplasmic plaque PDZ domain-containing proteins. It was reported that ZO-1 contributes to stabilizing claudin strands with the actin cytoskeleton [33]. In our research, ZO-1 and Claudin-1 were reduced in the sepsis mouse while their expressions were promoted by SST treatment. This result suggested that SST had a protective function in cell tight junction and barrier function.

In this current research, we also found that SST prevented intestinal barrier dysfunction with reduction of TNF-*α* and IL-6 and increase of IL-10 in the ileum in mice with sepsis, suggesting that SST might be potentially applied to the treatment of sepsis-induced intestinal barrier dysfunction through regulating inflammatory response progress. SST is released from the sensory nerves and activated by tissue damage and inflammatory mediators. It is known that specific somatostatin receptor subtype (SSTRs) may regulate the inflammatory pathway in the intestinal inflammation model [34]. The specific SST receptor SSTR5 in the intestinal barrier was detected increased by SST in mice with sepsis in this research, so we inferred that SST/SSTRs probably activated the anti-inflammation pathway in septic mouse.

It is known that the NF-*κ*B pathway plays a crucial role in CLP-induced sepsis [35]. Thus, we detected the expression of NF-*κ*B in the CLP-induced sepsis model in the presence of SST or not. Our findings indicated that NF-*κ*B p65 was promoted in the ileum by sepsis but inhibited by SST. JSH-23 is known to be an inhibitor of the NF-*κ*B pathway [36, 37]. Further experiments confirmed that JSH-23 could repair the intestinal barrier dysfunction, enhance the protective effect of SST on the intestinal barrier, and show an anti-inflammatory effect. Those results indicated that the inhibition of NF-*κ*B may be a mechanism underlying the protective role of SST in sepsis-induced intestinal barrier dysfunction.

In conclusion, our results found that mice with sepsis showed an obvious intestinal barrier dysfunction with an increase of TNF-*α*, IL-6, and IL-10 in the ileum. More importantly, SST has a protective effect on intestinal barrier dysfunction through the suppression of NF-*κ*B. Thus, SST could be a potential therapeutic drug for sepsis-induced intestinal barrier dysfunction.

## Data Availability

The analyzed data sets generated during the study are available from the corresponding author on reasonable request.
